# Tacrolimus Intrapatient Variability After Switching From Immediate or Prolonged-Release to Extended-Release Formulation, After an Organ Transplantation

**DOI:** 10.3389/fphar.2021.602764

**Published:** 2021-10-07

**Authors:** Arnaud Del Bello, Clotilde Gaible, Nathalie Longlune, Anne-Laure Hebral, Laure Esposito, Peggy Gandia, Nassim Kamar

**Affiliations:** ^1^ Department of Nephrology and Organ Transplantation, CHU Rangueil, Toulouse, France; ^2^ INSERM U1043, IFR–BMT, CHU Purpan, Toulouse, France; ^3^ Université Paul Sabatier, Toulouse, France; ^4^ Pharmacokinetics and Toxicology Laboratory, Toulouse University Hospital, Toulouse, France; ^5^ INTHERES, INRAE, ENVT, Université de Toulouse, Toulouse, France

**Keywords:** tacrolimus variability, solid organ transplantation, tacrolimus formulation, extended-release tacrolimus, outcomes, rejection

## Abstract

**Background and Purpose:** Several formulations of tacrolimus are available, but evidence of the benefit of changing to the most recent formulations is lacking. Tacrolimus intra-patient variability (tacrolimus IPV) is an emerging risk factor associated with poor graft outcomes after solid organ transplantations. Here, we examined the modifications of tacrolimus IPV after switching to a different formulation of tacrolimus.

**Experimental Approach:** We identified 353 solid organ transplant recipients that were switched in our center from immediate-release (IR-tacrolimus) or prolonged-release tacrolimus (PR-tacrolimus) to extended-release, LCP-tacrolimus (LCP-tacrolimus). Among them, 54 patients underwent at least 3 available tacrolimus blood concentrations before and after the switch, allowing us to investigate tacrolimus IPV.

**Key Results:** The switch was considered as a safe procedure since only four of the 353 patients presented a graft rejection after the switch, and no patient was hospitalized for tacrolimus overdose. The tacrolimus IPV estimated by the coefficient of variation (CV-IPV) was stable before and after the switch to LCP-tacrolimus (CV-IPV: 29.0% (IQR 25–75 (15.5; 38.5) before and 24.0% (15.8; 36.5) after the switch, p = 0.65).

**Conclusion **and Implications**:** Switching from IR- or PR-tacrolimus to LCP-tacrolimus is a safe procedure. However, the CV-tacrolimus IPV was not impacted by the change of formulation.

## Introduction

Tacrolimus is currently the cornerstone of immunosuppressive therapy after solid organ transplantations (Hart et al., 2019). However, it is characterized by a narrow therapeutic index, and a large interpatient variability (Kuypers, 2020). Furthermore, tacrolimus is also associated with intrapatient variability (IPV), requiring frequent measurements of tac concentration. A high IPV is an emerging risk factor after solid organ transplantations. It was suggested that IPV for tacrolimus may lead to periods of underexposure and overexposure, thus causing immune complications or drug-related toxicity. A high IPV was previously associated with poorer graft survival, the occurrence of *de novo* donor specific antibodies (DSAs), acute rejections, or calcineurin-inhibitors associated with kidney histological lesions (Sapir-Pichhadze et al., 2014; O’Regan et al., 2016; Rodrigo et al., 2016; Shuker et al., 2016; Vanhove et al., 2016; Goodall et al., 2017; Rahamimov et al., 2019).

Tacrolimus is available in several formulations: twice-daily immediate-release (IR-tacrolimus, initially Prograf^®^, Astellas Pharma, Tokyo, Japan, and thereafter generics), and more recently two once-daily formulations: a prolonged-release (PR-tacrolimus) formulation (Advagraf^®^, Astellas Pharma, Tokyo, Japan), and an extended-release (LCP-tacrolimus) formulation (Envarsus®, Chiesi Farmaceutici S. p.A, Parma, Italy). While the efficacy of these different formulations was confirmed by several previous studies (Barraclough et al., 2011; Tremblay et al., 2017), LCP-tacrolimus was associated with less fluctuations (defined as the ratio of the peak concentration minus the trough concentration over the average concentration) (Tremblay et al., 2017; Kamar et al., 2019). Tremblay and colleagues also previously demonstrated a higher exposure on a per milligram basis under LCP-tacrolimus comparing with IR- or PR-tacrolimus (Tremblay et al., 2017). This difference is explained by the use of Meltdose^®^ technology which improves the solubility of tacrolimus allowing for better oral availability by dispersing tacrolimus in a polymeric matrix, and a progressive resorption of tac throughout the digestive tract (Grinyó and Petruzzelli, 2014). Nonetheless, the decrease in fluctuation was not associated with improved outcomes after transplantations, until now.

Tacrolimus formulation is considered as one of the causes of tacrolimus IPV. However, only scarce data exist regarding the impact of switching to LCP-tacrolimus on the tacrolimus IPV after solid organ transplantations for patients who previously received IR-tacrolimus or PR-tacrolimus. In this study, we retrospectively assessed the tacrolimus IPV in a cohort of heart, liver, lung, and kidney transplant recipients having converted to LCP-tacrolimus.

### Patients and Method

All solid organ transplant patients having converted from IR-tacrolimus (Prograf®, Astellas Pharma, Tokyo, Japan) or PR-tacrolimus (Advagraf®, Astellas Pharma, Tokyo, Japan) to LCP-tacrolimus (Envarsus®, Chiesi Farmaceutici, Parma, Italy) between September 01, 2015 and February 01, 2020 were included in this retrospective study (*n* = 353). The patients’ characteristics are set forth in Table 1. Moreover, patient’s characteristics of the 54 patients in which the CV-IPV were assessed was detailed in Table 2.

**TABLE 1:** Mains characteristics of the patients included in the tacrolimus CV-IPV change after the switch to LCP-tacrolimus.

Variable	Number of patients (*n* = 54) (%)
Transplanted organ	
Kidney	30 (55.6)
Liver	20 (37.0)
Combined liver and kidney	3 (5.6)
Combined pancreas and kidney	1 (1.8)
Recipients’ gender, male	32 (59)
Recipients’ age, years mean ± SD	58 ± 16
Time between transplantation—switch to LCP-tac, months median (IQR_25-75_)	23.5 (12.0–43.8)
Cause of switch to LCP-tac	
side effects (tremor/digestive trouble)	16 (29.6)
Local practice	38 (70.4)
Associated Immunosuppression regimen	
MPA	47 (87.0)
Everolimus	3 (5.6)
Azathioprine	1 (1.9)
Steroids	49 (90.7)
Leflunomide	0
Tacrolimus monotherapy	1 (1.8)
Tacrolimus bitherapy with	
steroids	3 (5.6)
MPA	1 (1.8)
Everolimus	2 (3.7)
Tacrolimus tritherapy with	
MPA and steroids	45 (83.5)
everolimus and MPA	1 (1.8)
azathioprine and steroids	1 (1.8)
Tacrolimus trough level	
before the switch, mean ± SD	6.9 ± 2.0
after the switch, mean ± SD	6.7 ± 1.8
Tacrolimus formulation before the switch	
IR-tacrolimus	
IR-tac blood concentration, ng/mL, mean ± SD	7.2 ± 2.1
IR-tac dose, mg/day, median (IQR_25-75_)	6.0 (5.7–7.7)
PR-tacrolimus	
PR-tac blood concentration, ng/mL, mean ± SD	6.2 ± 1.8
PR-tac dose, mg/day, median (IQR_25-75_)	5.5 (5.4–7.7)
LCP-tacrolimus dose, mg/day, median (IQR_25-75_)	3.4 (2.0–6.0)

LCP-tacrolimus, once daily extended release tacrolimus; IR, immediate release; PR, prolonged release; MPA, mycophenolic acid; SD, standard deviation.

**TABLE 2:** Mains characteristics of the patients converted to LCP-tacrolimus.

Variables	Number (%) *n* = 353
Transplanted organ	
Kidney	232 (65.6)
Liver	86 (24.3)
Heart	12 (3.4)
Lung	3 (0.8)
Pancreas	2 (0.6)
Combined	
Liver-kidney	16 (4.4)
Heart-kidney	1 (0.3)
Pancreas-kidney	1 (0.3)
Lung-kidney	1 (0.3)
Recipients’ age, years, mean ± SD	55.5 ± 11.9
Recipients’ gender, male	214 (60.6)
Time from transplantation to conversion to LCP-tacrolimus, months, median (IQR_25-75_)	36 (10–90)
Cause of switch	
side effects	94 (26.6)
Local practice	259 (73.4)
Associated Immunosuppression regimen	
MPA	300 (84.5)
everolimus	25 (7)
azathioprine	5 (1.4)
belatacept	1 (0.3)
leflunomide	1 (0.3)
steroids	315 (89.2)
Tacrolimus monotherapy (%)	14 (4.0)
Tacrolimus bitherapy with	
steroids	26 (7.0)
MPA	43 (12.2)
everolimus	6 (2.0)
leflunomide	1 (0.3)
azathioprine	1 (0.3)
Tacrolimus tritherapy with	
MPA and steroids	189 (53.5)
everolimus and steroids	64 (18.1)
azathioprine and steroids	7 (2.0)
MPA and everolimus	1 (0.3)
Everolimus and azathioprine	1 (0.3)
Serum creatinine (µmol/L), median (IQR_25-75_)	
1 year before conversion	130.0 (100.0–155.5)
at conversion	135.0 (105.0–150.0)
1 year after conversion	130.0 (104.5–163.0)

LCP-tacrolimus, once daily extended release tacrolimus; MPA, mycophenolic acid; SD, standard deviation.

The cause of switch was identified on the basis of the patient record, and separated between 1) the side effect supposed to be related to tacrolimus formulation, and 2) a switch proposed by the transplant physician, due to modification of local practice in the number one choice of tacrolimus formulation.

The time from transplantation to conversion to LCP-tacrolimus was 36 (3–222) months in the total population, and 23.5 (12–44) months in the 54 patients in which CV-IPV was calculated.

All the patients followed an educational program during the first week after transplantation, concerning the timing of tacrolimus dosing, tacrolimus dosing and food intake, and the need to avoid herbal preparations or treatments classically associated with tacrolimus blood exposure interferences. During the follow-up after transplantation, additional sessions were proposed at the request of the clinician.

The switch to LCP-tacrolimus was performed with a dosage conversion factor of 1:0.7, from 1 day to the next. This conversion factor was applied by taking the total daily dose in patients under IR-tacrolimus or PR-tacrolimus.

In accordance to the French Ethics Law, the patients were informed that their codified data would be used for the study. According to the French Ethics and Regulatory Law (Public Health Code), retrospective studies based on the exploitation of usual care data are not required to be submitted to an Ethics Committee but they must be declared or covered by the reference methodology of the French National Commission for Informatics *and Liberties (CNIL).* The collection and computer processing of personal and medical data were implemented in order to analyze the results of the research. Toulouse University Hospital signed a Commitment of Compliance to Reference Methodology MR-004 of the French National Commission for Informatics and Liberties (CNIL). After evaluation and validation by the Data Protection Officer and in accordance with the General Data Protection Regulation, this study was deemed as meeting all the criteria, was recorded in the Register of Retrospective Studies of the Toulouse University Hospital (Register number: *RnIPH 2020-84*) and covered by the MR-004 CNIL Methodology (CNIL number: 2206723 v 0). This study was approved by Toulouse University Hospital and it was further confirmed that all ethical requirements were met in the above report.

### Immunosuppressive Regimen

The tacrolimus target of the trough concentration (i.e., the concentration measured in a blood sample collected just before drug administration) was maintained in the 8–10 ng/ml range during the first year and then maintained in the 5–7 ng/ml thereafter, when associated with mycophenolic acid (MPA), leflunomide, or azathioprine. The tacrolimus target was adjusted in the 5–7 ng/ml range, when associated with everolimus (with a target of 3–5 ng/ml).

Patients received the same association of immunosuppressive treatments before and after the switch to LCP-tacrolimus.

In this cohort, no patients received a generic tac substitution.

### Intrapatient Variability

Tacrolimus trough concentrations were measured in blood samples using a validated liquid chromatography-tandem mass spectrometry presenting a within-day and between-days precision of less than 15%, as previously described (Saint-Marcoux et al., 2011). Tacrolimus concentrations available at months 12, 9, 6, and 3 before and on the date of the switch to LCP-tac, and then at months 1, 3, 6, 9, and 12 after the date of the switch were analyzed.

Only patients with at least 3 tac C0 before and 3 tac C0 after the switch were included in the CV-IPV analysis.

As previously described (Kuypers, 2020), to avoid confounding factors associated with tac exposure during this period (e.g., the decrease of steroid doses, or the impact of anemia), the tacrolimus concentrations obtained during the first 3 months posttransplantation, as well as those obtained during hospitalization (except for outpatient admissions), were excluded.

To assess tacrolimus IPV before and after the switch, we excluded patients who were converted to LCP-tacrolimus before 6 months posttransplantation (n = 100). For those converted at months six posttransplant, or thereafter, only trough levels obtained after 3 months posttransplantation were considered for the calculation of tacrolimus IPV before the conversion.

Tacrolimus blood concentrations higher than 20 were reviewed and excluded if a doubt existed regarding the validity of the sampling time (i.e., when the blood sample was collected after the theoretical time of drug administration, the measured concentration was excluded as this value was likely located in the absorption phase). Moreover, included concentrations were considered to be at steady state as a 3-day interval was imposed between whatever drug-dosage modification and blood sampling.

We used the coefficient of variation (CV) to compare the intrapatient variability before and after the switch to LCP-tacrolimus. The CV-IPV was calculated as follows: CV-IPV (%) = (standard deviation/mean tac trough-level concentration) × 100.

### Immunological Analyses

The presence of *de novo* DSAs was investigated systematically at months 3 and 12 posttransplant, and 1 year after the switch to LCP-tacrolimus. Furthermore, additional anti-HLA DSA testing was performed in the event of graft dysfunction, and suspicions of graft rejection. The presence of DSAs was tested using the Labscreen™ Single Antigen technology (One Lambda, Canoga Park, CA). The Labscreen™ Single Antigen determined the specificity of Class I HLAs in A/B/Cw and Class II in DR/DQ/DP IgG antibodies in the recipients' sera (centrifuged at 10,000 *g* for 10 min) as per the manufacturer’s instructions. The presence and specificity of the antibodies were then detected using a Labscan 100®, and the mean fluorescence (baseline value) for each sample in each bead was evaluated. A baseline mean fluorescence intensity value of >1,000 was considered positive.

### Pathological Analysis

All the rejection episodes were biopsy-proven, and classified according to the adapted Banff classification (Demetris et al., 1997; Demetris et al., 2000; Drachenberg et al., 2011; Haas et al., 2014; Demetris et al., 2016; Bruneval et al., 2017; Haas et al., 2018).

### Statistical Analysis

The reported values represent the means (±SD) or medians (ranges) when appropriate. Proportions were compared using the chi-squared, or Fisher’s exact test when necessary. Quantitative variables were compared using the Mann–Whitney nonparametric test. A *p*-value of <0.05 was considered statistically significant. All the statistical analyses were performed on GraphPad PRISM v8.0 or XLSTAT v19.01.

## Results

### CV-IPV Evaluation Before and After the Switch

**FIGURE 1:**
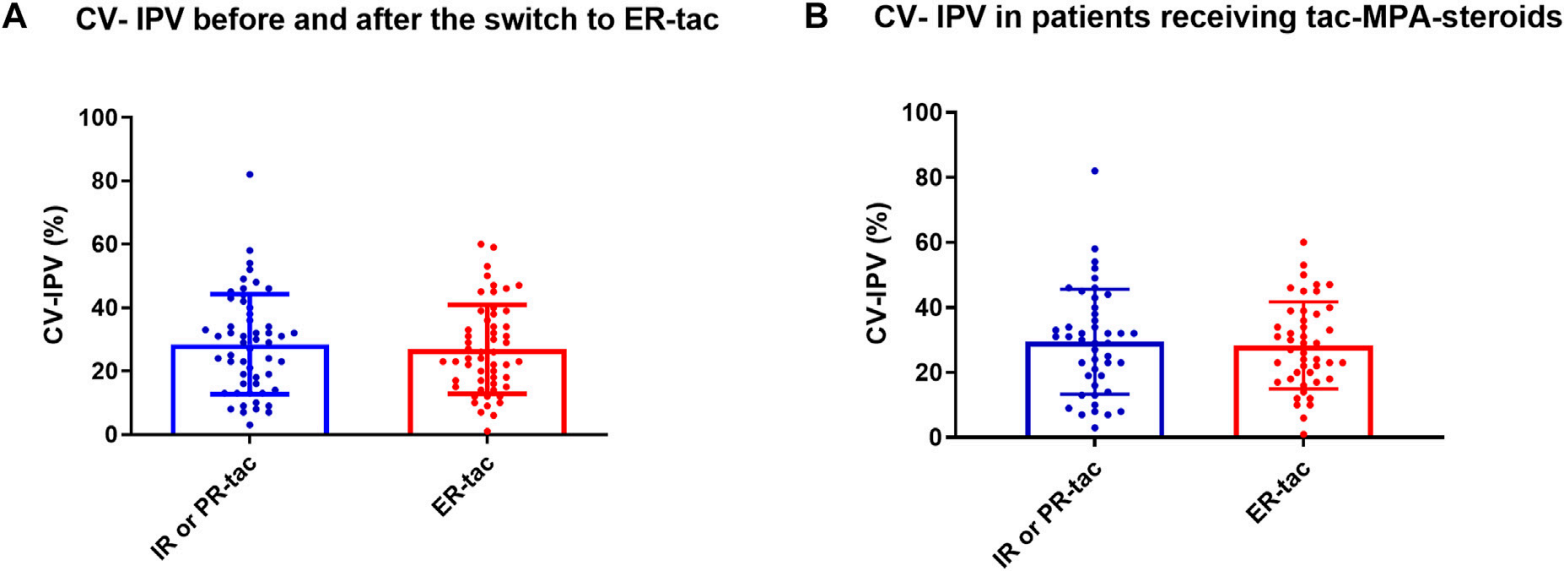
**(A-B)**. CV-tacrolimus IPV in patients treated with IR or PR-tacrolimus and converted to LCP-tacrolimus. IR-tac, immediate-release tacrolimus; PR-tac, prolonged-release tacrolimus; LCP-tac, extended-release LCP-tac; MPA, Mycophenolic acid. Results are presented as mean with SD.

During the follow-up, 54 patients underwent at least 3 available tac blood concentrations, before and after the switch (Table 1). The mean number of tac C0 included in the analysis was 4 ± 0.7 before, and 4 ± 0.7 after the switch. The median CV-IPV was of 29.0% (IQR _25–75_ (15.5–38.5)) before the switch and of 24.0% (15.8–36.5) after (*p* = 0.65) (Figure 1A). Twenty-six of the 54 (48%) presented a higher CV-IPV with LCP-tacrolimus in comparison to the results obtained before the change of formulation. We did not find any difference concerning the transplanted organ, patient age, gender, reason leading to conversion, time between transplantation and conversion, immunosuppressive strategy, or renal function, in these patients comparing with those for which CV-IPV decreased post switch (Additional Supplementary Table S1). The CV-IPV was unchanged before and after the conversion in the 45 patients who were given triple therapy by tac-MPA and steroids (30% (IQR _25–75_ (17.5–39.0) and 27% (IQR _25–75_ (18.0–38.5), *p* = 0.84) (Figure 1B). The CV-IPV was unchanged before and after the conversion in the 14 patients who received PR-tacrolimus (29.0% (IQR _25–75_ (18.0–42.0)) and 23% (IQR _25–75_ (14.0–34.0), *p* = 0.63), as well as in the 40 patients who received IR-tacrolimus (29 (13–38) and 24% (IQR _25–75_ (17–39), *p* = 0.79).

The CV-IPV was similar in kidney transplant patients (28.0% (IQR _25–75_ (18.3–33.3)) before and 25.5% (IQR _25–75_ (19.5–38.5)) after, *p* = 0.88), or liver transplant patients (26.0% (IQR _25–75_ (13.0–41.0)) before and 24.5% (IQR _25–75_ (12.5–33.5)), *p* = 0.70). No statistical difference was observed when comparing the CV-IPV according to the transplanted organ, kidney or liver (*p* = 0.90 before the conversion, and *p* = 0.38 after the conversion). The CV-IPV was not influenced by recipient sex, serum creatinine, or recipient age (additional Figure 2).

**FIGURE 2:**
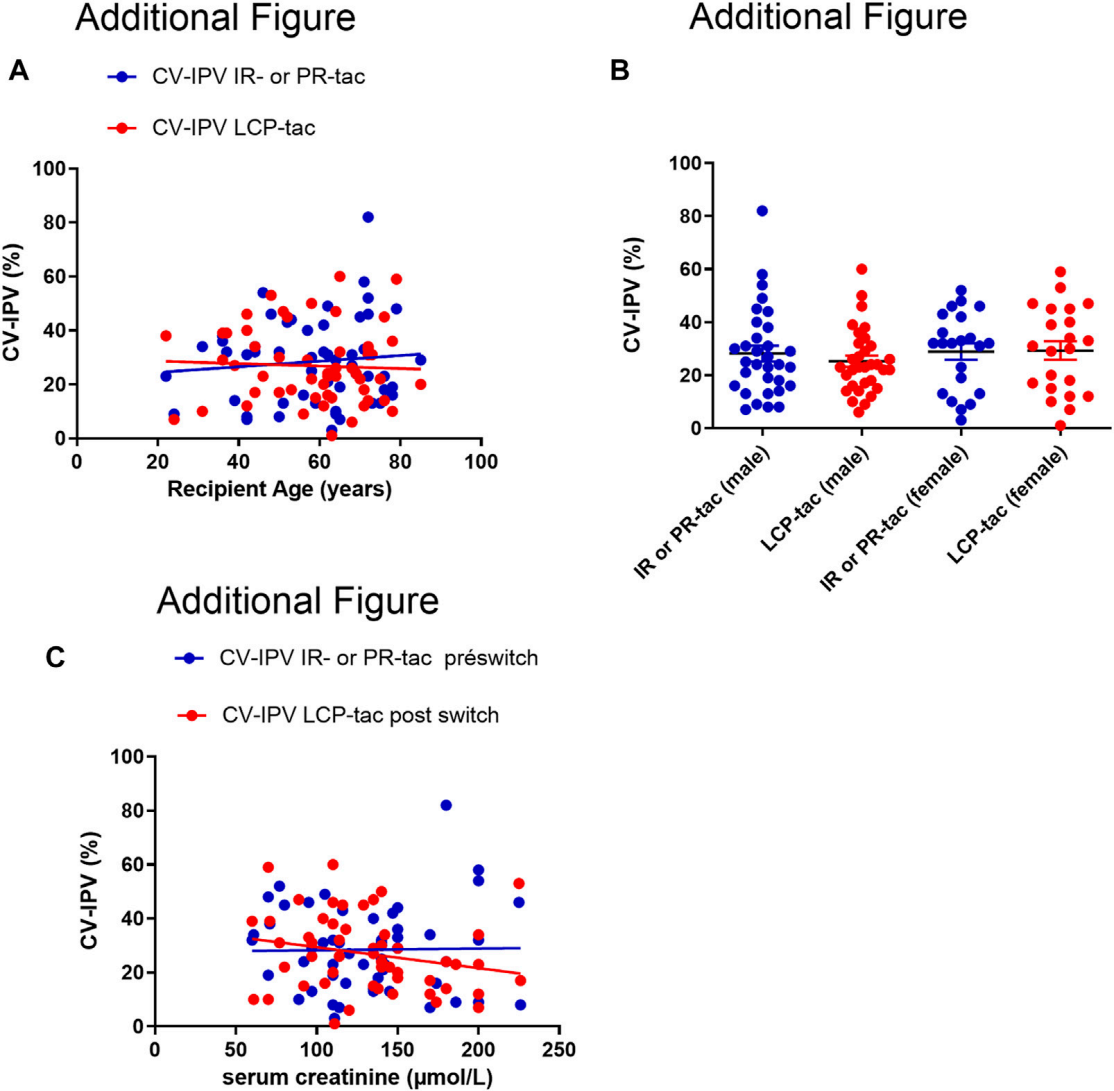
**(A)**: Correlation between CV-IPV (%) and recipient age, before and after conversion to ER-LCP tacrolimus (the Pearson correlation was *r*= 0.0009, *p*=0.48 for IR our PR-tac, and *r*=0.002, *p*=0.73 with ER-LCP tacrolimus). **(B)**: CV-tac IPV according to the recipient gender before and after the switch to ER-LCP tacrolimus. IR-tac, Immediate-Release tacrolimus; PR-tac, Prolonged-Release tacrolimus; ER-tac, Extended-Release LCP-tac Results are presented as mean with SD. **(C)**: Correlation between CV-IPV (%) and serum creatinine (μmol/L), before and after conversion to ER-LCP tacrolimus (the Pearson correlation was *r*=0.0002, *p*=0.90 for IR our PR-tac, and *r*=0.05, *p*=0.10 with ER-LCP Tacrolimus).

The CV-IPV was 36.0% (IQR _25–75_ (31.0–44.0) before the switch and 29.0% (IQR _25–75_ (22.0–39.0)) after in patients switched during the first year posttransplant (*p* = 0.20). For the patients switched after 1 year the CV-IPV was 24.0% (IQR _25–75_ (13.0–34.0) before and 23.0% (IQR _25–75_ (15.0–36.0) after the switch (*p* = 0.96). To note the CV-IPV before the switch was significantly higher in patients that were switched before 1 year than the others (36.0% (IQR _25–75_ (31.0–44.0) in patients switched before 1 year, and 24.0% (IQR _25–75_ (13.0–34.0), in patients switched after 1 year, *p* = 0.03).

### *The Switch From IR- or PR-Tac*rolimus *to LCP-Tac*rolimus *Is Safe*

A switch from IR-tacrolimus (*n* = 234, 66%) or PR-tacrolimus (*n* = 119, 34%) to LCP-tacrolimus was performed in 353 patients (Table 1). Sixty-seven (19%) patients were converted during the first 6 months posttransplant, and 101 (28.6%) during the first year posttransplant. The reason of tacrolimus formulation change was mainly driven by a local protocol (*n* = 262), or motivated by tacrolimus side effects (tremor in 80 patients, headaches and behavior changes in seven patients, and digestive disorders in seven patients).

The two-thirds of the cohort were kidney transplant recipients: eighty-six (37%) were from living donors, 35 (15%) and 47 (20%) were ABO-incompatible kidney transplants and HLA-incompatible kidney transplants, respectively.

**FIGURE 3:**
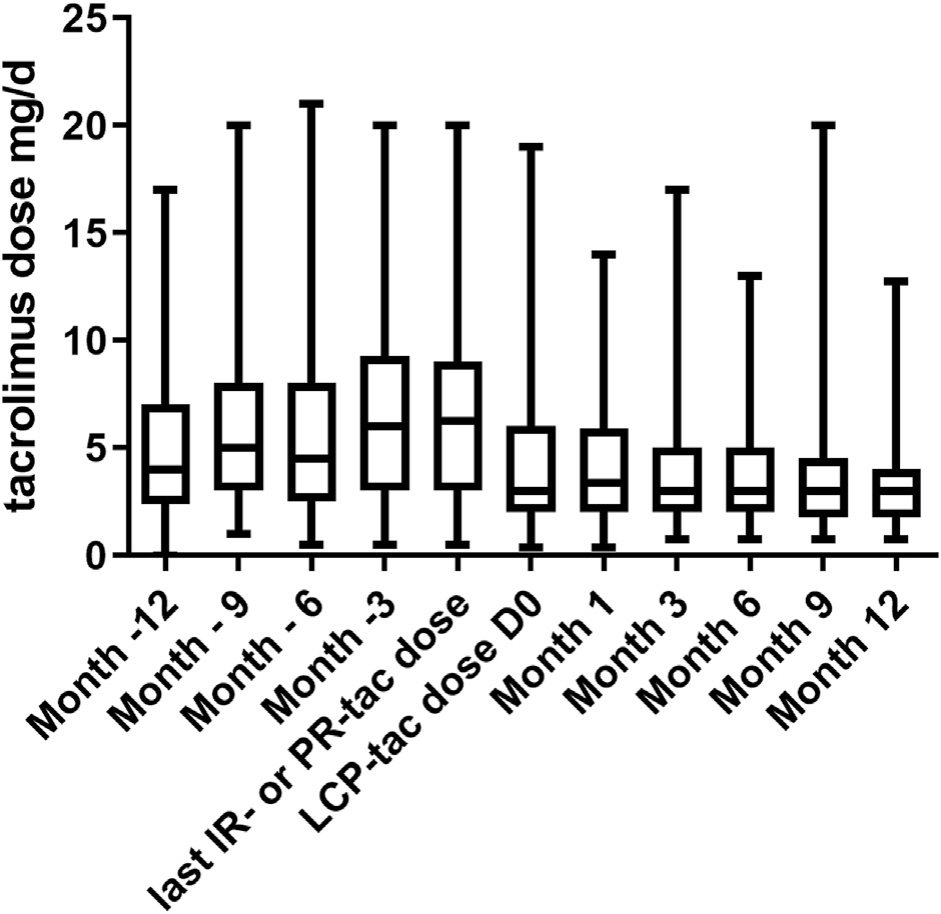
Evolution of tacrolimus dose (mg/d) during the year before and after the switch from IR-tacrolimus or PR-tac to LCP-tacrolimus. IR-tac, immediate-release tacrolimus; PR-tac, prolonged-release tacrolimus; LCP-tac, extended-release LCP-tac. Results are presented as mean with SD.

The evolution of tac posology before and after the switch is set forth in Figure 3. As expected, the tacrolimus dose was lower with LCP-tac in comparison to IR-tac or PR-tac (median dose 6.5 (IQR_25-75_ (5.3–8.3)) mg/d and 3 (IQR_25-75_ (2.0–4.8)) mg/d before and after the switch, respectively, *p* < 0.0001). The initial posology of LCP-tacrolimus was unchanged at month one in 288 (81.6%) patients. No patient was hospitalized for a tacrolimus overdose during the year following the switch.

During the first year after the conversion to LCP-tacrolimus, four patients (1.1%) presented a biopsy-proven acute rejection. Two liver transplant recipients presented a moderate (n = 1) or severe (=1) T cell–mediated rejection, respectively, 4 and 3 months after the switch (16 and 9 months posttransplant). One heart-transplant recipient presented a moderate T cell–mediated rejection, 1 month after the switch (10 months posttransplant). One kidney transplant recipient presented a mixed rejection with an occurrence of *de novo* DSA 3 months after the switch, and 9 months posttransplant. It should be noted that all these patients were previously on IR-tacrolimus, and the switch to LCP-tacrolimus was motivated by tacrolimus blood concentration variability in 3 of the four patients.

Four recipients (1.1%) presented *de novo* DSAs after the switch to LCP-tacrolimus: two kidney-transplant recipients presented anti-class II DSAs 3 and 8 months post switch (11 and 9 months posttransplant), and two liver transplant recipients presented anti-class II DSAs, 4 and 8 months post switch (16 and 36 months posttransplant).

Thirty-two (9%) patients stopped LCP-tacrolimus less than 1 year after they started the treatment: 21 patients presented neurological (n = 16) or digestive (n = 5) adverse events, six patients presented a tacrolimus blood concentration higher than the target with the lowest posology available, 3 patients were switched to a CNI belatacept-free based regimen because of an impaired kidney function, and two patients lost their graft.

## Discussion

In this study, we found that 1) tacrolimus variability was stable for a majority of the patients before and after the switch from IR- or PR-tacrolimus to LCP-tacrolimus, and 2) the switch from IR-tacrolimus or PR-tacrolimus to LCP-tacrolimus is safe and well-tolerated.

Tacrolimus IPV was recently introduced as a promising clinical marker, associated with graft survival, the occurrence of acute rejections, the occurrence of *de novo* DSAs, or chronic immune-related lesions after kidney (Rodríguez-Perálvarez et al., 2012; Goodall et al., 2017; Rahamimov et al., 2019), heart (Gueta et al., 2018), lung (Gallagher et al., 2015), and liver transplants (Bello et al., 2018). Tacrolimus formulation is one of the main factors classically involved in tacrolimus IPV, such as nonadherence, drug–drug interactions, or gastrointestinal disorders (Kuypers, 2020). In the present study, we found that tacrolimus-IPV was stable in a large majority of the patients under IR-, PR-, or LCP-tacrolimus. Recently, Leino and colleagues investigated the tacrolimus IPV in a cohort of stable and highly adherent liver or kidney transplant recipients. They found a median tacrolimus IPV of approximately 15 (4.8–110) % and did not find any difference when comparing the CV per transplant type or IR-tacrolimus formulation. Furthermore, they did not find any factors associated with a high CV-IPV (>30%). Our results are consistent with this study, with a CV-IPV 29.0% (IQR _25–75_ (15.5; 38.5)) before the switch and of 24.0% (15.8; 36.5) after (*p* = 0.65). Hence, other approaches to reduce tacrolimus IPV should be considered rather than formulation changes. Since other modifiable determinants of IPV are represented by food (mainly driven by timing and fat content) and drug interaction, but also by medical nonadherence, a regular therapeutic education could improve this point. Future studies to confirm this point are required. Moreover, genetic factors (CYP3A5 polymorphisms) have been associated with tacrolimus variability, and elaborating future studies targeting specific actions to reduce variability among CYP3A5 expressers are needed.

The safety of conversion to LCP-tacrolimus was previously investigated in a prospective, multicenter, phase III, non-inferiority study (Bunnapradist et al., 2013). The authors demonstrated in a cohort of 326 kidney transplant patients similar efficacy and failure rates between patients converted to LCP-tacrolimus and those who were maintained under IR-tacrolimus (Bunnapradist et al., 2013). Recently, Sánchez-Fructuoso and colleagues (Sánchez Fructuoso et al., 2020) investigated the safety of switching from IR- or PR-tacrolimus to LCP-tacrolimus in a retrospective cohort of 365 kidney transplant patients. Three months after the switch, no occurrence of biopsy-proven acute rejection, or kidney function impairment were reported (Sánchez Fructuoso et al., 2020). This is in line with our results, since in this large cohort of solid organ transplant recipients, 1 year after the switch only four patients developed an acute rejection, and four patients developed *de novo* DSAs.

This study has several limitations. First, it is a retrospective, non-randomized, observational single-center study. Although 353 patients were switched from IR-tacV or PR-tacrolimus to LCP-tacrolimus, only 54 patients were included in the analyses of tacrolimus IPV. This can be considered as an important limitation of the study. Nevertheless, we have chosen to analyze homogenous data by including the 54 patients who had undergone six trough-level measurements (3 before and 3 after the switch) in the same laboratory and with the same assay to avoid adding a confounding factor. In this homogenous population, we did not observe a significant modification in CV-IPV after the switch from the switch from IR- or PR-tacrolimus to LCP-tacrolimus. Our data should be confirmed in a larger cohort. Second, some other factors that can influence IPV have not been assessed in the retrospective study, that is, nonadherence (Vanhove et al., 2016), hematocrit level, and CYP3A4 and CYP3A5 genotyping. Nevertheless, since no specific intervention, such as therapeutic education, was performed after conversion from one formulation to another and since each patient was his own control, it is unlikely that these parameters could have changed our results. Third, unfortunately, we did not assess the quality of life after the switch. Fourth, due the small number of patients and relatively short follow-up, we were not able to assess the clinical impact of the reduction of IPV in patients with initially high CV-IPV. Further studies are required to assess the clinical outcome according to the modification of CV-IPV.

In conclusion, the switch from IR-tacrolimus or PR-tacrolimus to LCP-tacrolimus is safe and well-tolerated. In a small homogenous cohort of patients, the CV-tacrolimus IPV remained stable after the conversion from IR or PR to LCP-tacrolimus formulation.

## Data Availability

The raw data supporting the conclusion of this article will be made available by the authors, without undue reservation.

## Abbreviations

Intrapatient variability, IPV; DSA, donor-specific antibodies; IR-tac, immediate-release tacrolimus; PR-tac, prolonged-release tacrolimus; LCP-tac, once-daily extended-release tacrolimus; MPA, mycophenolic Acid.

## References

[B1] Barraclough K. A. Isbel N. M. Johnson D. W. Campbell S. B. Staatz C. E. (2011). Once- versus Twice-Daily Tacrolimus. Drugs 71 (12), 1561–1577. 10.2165/11593890-000000000-00000 21861541

[B2] Del Bello A. Congy-Jolivet N. Danjoux M. Muscari F. Lavayssière L. Esposito L. (2018). High Tacrolimus Intra-patient Variability Is Associated with Graft Rejection, and De Novo Donor-specific Antibodies Occurrence after Liver Transplantation. Wjg 24 (16), 1795–1802. 10.3748/wjg.v24.i16.1795 29713132 PMC5922997

[B3] Bruneval P. Angelini A. Miller D. Potena L. Loupy A. Zeevi A. (2017). The XIIIth Banff Conference on Allograft Pathology: The Banff 2015 Heart Meeting Report: Improving Antibody-Mediated Rejection Diagnostics: Strengths, Unmet Needs, and Future Directions. Am. J. Transpl. 17 (1), 42–53. 10.1111/ajt.14112 PMC5363364 27862968

[B4] Bunnapradist S. Ciechanowski K. West‐Thielke P. Mulgaonkar S. Rostaing L. Vasudev B. (2013). Conversion from Twice‐Daily Tacrolimus to Once‐Daily Extended Release Tacrolimus (LCPT): The Phase III Randomized MELT Trial. Am. J. Transplant. 13 (3), 760–769. 10.1111/ajt.12035 23279614 PMC3613750

[B5] Demetris A. Adams D. Bellamy C. Blakolmer K. Clouston A. Dhillon A P. (2000). Update of the International Banff Schema for Liver Allograft Rejection: Working Recommendations for the Histopathologic Staging and Reporting of Chronic Rejection. Hepatology 31, 792–799. 10.1002/hep.510310337 10706577

[B6] Demetris A. J. Batts K. P. Dhillon A. P. Ferrell L. Fung J. Geller S. A. (1997). Banff Schema for Grading Liver Allograft Rejection: An International Consensus Document. Hepatology 25 (3), 658–663. 10.1002/hep.510250328 9049215

[B7] Demetris A. J. Bellamy C. Hübscher S. G. O'Leary J. Randhawa P. S. Feng S. (2016). Comprehensive Update of the Banff Working Group on Liver Allograft Pathology: Introduction of Antibody-Mediated Rejection. Am. J. Transpl. 16, 2816-2835. 10.1111/ajt.13909 27273869

[B8] Drachenberg C. B. Torrealba J. R. Nankivell B. J. Rangel E. B. Bajema I. M. Kim D. U. (2011). Guidelines for the Diagnosis of Antibody-Mediated Rejection in Pancreas Allografts-Updated Banff Grading Schema. Am. J. Transpl. 11, 1792–1802. 10.1111/j.1600-6143.2011.03670.x 21812920

[B9] Gallagher H. M. Sarwar G. Tse T. Sladden T. M. Hii E. Yerkovich S. T. (2015). Erratic Tacrolimus Exposure, Assessed Using the Standard Deviation of Trough Blood Levels, Predicts Chronic Lung Allograft Dysfunction and Survival. J. Heart Lung Transplant. 34 (11), 1442–1448. 10.1016/j.healun.2015.05.028 26186804

[B10] Goodall D. L. Willicombe M. McLean A. G. Taube D. (2017). High Intrapatient Variability of Tacrolimus Levels and Outpatient Clinic Nonattendance Are Associated with Inferior Outcomes in Renal Transplant Patients. Transplant. Direct 3 (8), e192. 10.1097/txd.0000000000000710 28795143 PMC5540630

[B11] Grinyó J. M. Petruzzelli S. (2014). Once-daily LCP-Tacro MeltDose Tacrolimus for the Prophylaxis of Organ Rejection in Kidney and Liver Transplantations. Expert Rev. Clin. Immunol. 10 (12), 1567–1579. 10.1586/1744666X.2014.983903 25407098

[B12] Gueta I. Markovits N. Yarden-Bilavsky H. Raichlin E. Freimark D. Lavee J. (2018). High Tacrolimus Trough Level Variability Is Associated with Rejections after Heart Transplant. Am. J. Transpl. 18 (10), 2571–2578. 10.1111/ajt.15016 29989311

[B13] Haas M. Loupy A. Lefaucheur C. Roufosse C. Glotz D. Seron D. (2018). The Banff 2017 Kidney Meeting Report: Revised Diagnostic Criteria for Chronic Active T Cell-Mediated Rejection, Antibody‐mediated Rejection, and Prospects for Integrative Endpoints for Next‐generation Clinical Trials. Am. J. Transpl. 18, 293–307. 10.1111/ajt.14625 PMC5817248 29243394

[B14] Haas M. Sis B. Racusen L. C. Solez K. Glotz D. Colvin R. B. (2014). Banff 2013 Meeting Report: Inclusion of C4d-Negative Antibody-Mediated Rejection and Antibody-Associated Arterial Lesions. Am. J. Transpl. 14, 272–283. 10.1111/ajt.12590 24472190

[B15] Hart A. Smith J. M. Skeans M. A. Gustafson S. K. Wilk A. R. Castro S. (2019). OPTN/SRTR 2017 Annual Data Report: Kidney. Am. J. Transpl. 19, 19–123. 10.1111/ajt.15274 30811893

[B16] Kamar N. Cassuto E. Piotti G. Govoni M. Ciurlia G. Geraci S. (2019). Pharmacokinetics of Prolonged-Release Once-Daily Formulations of Tacrolimus in De Novo Kidney Transplant Recipients: A Randomized, Parallel-Group, Open-Label, Multicenter Study. Adv. Ther. 36 (2), 462–477. 10.1007/s12325-018-0855-1 30552587 PMC6824349

[B17] Kuypers D. R. J. (2020). Intrapatient Variability of Tacrolimus Exposure in Solid Organ Transplantation: A Novel Marker for Clinical Outcome. Clin. Pharmacol. Ther. 107 (2), 347–358. 10.1002/cpt.1618 31449663

[B18] O’Regan J. A. Canney M. Connaughton D. M. O’Kelly P. Williams Y. Collier G. (2016). Tacrolimus Trough-Level Variability Predicts Long-Term Allograft Survival Following Kidney Transplantation. J. Nephrol. 29 (2), 269–276. 10.1007/s40620-015-0230-0 26374111

[B19] Rahamimov R. Tifti-Orbach H. Zingerman B. Green H. Schneider S. Chagnac A. (2019). Reduction of Exposure to Tacrolimus Trough Level Variability Is Associated with Better Graft Survival after Kidney Transplantation. Eur. J. Clin. Pharmacol. 75 (7), 951–958. 10.1007/s00228-019-02643-y 30762079

[B20] Rodrigo E. Segundo D. S. Fernández-Fresnedo G. López-Hoyos M. Benito A. Ruiz J. C. (2016). Within-Patient Variability in Tacrolimus Blood Levels Predicts Kidney Graft Loss and Donor-specific Antibody Development. Transplantation 100 (11), 2479–2485. 10.1097/TP.0000000000001040 26703349

[B21] Rodríguez-Perálvarez M. Germani G. Darius T. Lerut J. Tsochatzis E. Burroughs A. K. (2012). Tacrolimus Trough Levels, Rejection and Renal Impairment in Liver Transplantation: A Systematic Review and Meta-Analysis. Am. J. Transpl. 12 (10), 2797–2814. 10.1111/j.1600-6143.2012.04140.x 22703529

[B22] Saint-Marcoux F. Debord J. Parant F. Labalette M. Kamar N. Rostaing L. (2011). Development and Evaluation of a Simulation Procedure to Take into Account Various Assays for the Bayesian Dose Adjustment of Tacrolimus. Ther. Drug Monit. 33 (2), 171–177. 10.1097/FTD.0b013e31820d6ef7 21383655

[B23] Sánchez Fructuoso A. Ruiz J. C. Franco A. Diekmann F. Redondo D. Calviño J. (2020). Effectiveness and Safety of the Conversion to MeltDose Extended‐release Tacrolimus from Other Formulations of Tacrolimus in Stable Kidney Transplant Patients: A Retrospective Study. Clin. Transpl. 34 (1). 10.1111/ctr.13767 PMC7050537 31815310

[B24] Sapir-Pichhadze R. Wang Y. Famure O. Li Y. Kim S. J. (2014). Time-dependent Variability in Tacrolimus Trough Blood Levels Is a Risk Factor for Late Kidney Transplant Failure. Kidney Int. 85 (6), 1404–1411. 10.1038/ki.2013.465 24336032

[B25] Shuker N. Shuker L. van Rosmalen J. Roodnat J. I. Borra L. C. P. Weimar W. (2016). A High Intrapatient Variability in Tacrolimus Exposure Is Associated with Poor Long-Term Outcome of Kidney Transplantation. Transpl. Int. 29 (11), 1158–1167. 10.1111/tri.12798 27188932

[B26] Tremblay S. Nigro V. Weinberg J. Woodle E. S. Alloway R. R. (2017). A Steady-State Head-To-Head Pharmacokinetic Comparison of All FK-506 (Tacrolimus) Formulations (ASTCOFF): An Open-Label, Prospective, Randomized, Two-Arm, Three-Period Crossover Study. Am. J. Transpl. 17 (2), 432–442. 10.1111/ajt.13935 PMC5297985 27340950

[B27] Vanhove T. Vermeulen T. Annaert P. Lerut E. Kuypers D. R. J. (2016). High Intrapatient Variability of Tacrolimus Concentrations Predicts Accelerated Progression of Chronic Histologic Lesions in Renal Recipients. Am. J. Transpl. 16 (10), 2954–2963. 10.1111/ajt.13803 27013142

