# Supplementary material for: Tacrolimus Intrapatient Variability After Switching From Immediate or Prolonged-Release to Extended-Release Formulation, After an Organ Transplantation
Source: Front Pharmacol. 2021 Oct 7;12:602764. doi: 10.3389/fphar.2021.602764 (PMC8529208; doi:10.3389/fphar.2021.602764)
Supplement: Supplementary file 1 [file Table1.docx]

Additional Table 1. Characteristics of recipients, according to the increase or not of the CV-IPV after the switch.

| Variables | Post switch decrease of  CV-IPV (n= 28) | Post switch increase of  CV-IPV (n= 26) | *p* |
| --- | --- | --- | --- |
| Transplanted organ   - Kidney - Liver - Combined:   Liver-kidney  Pancreas-kidney | 15 (53.6)  11 (39.3)  2 (7.1)  1 (3.6)  1 (3.6) | 15 (57.7)  9 (34.6)  2 (7.7)  2 (7.7)  0 | 0.94 |
| Recipients’ age (years), mean ± SD | 61 ± 14 | 56 ± 15 | 0.30 |
| Recipients’ gender, male | 17 (60.7) | 15 (57.7) | 0.89 |
| Time from transplantation to conversion to LCP-tacrolimus (months), median (IQR_25-75_) | 24 (12 – 60) | 24 (13 - 41) | 0.69 |
| Cause of switch (%)   - side effects - Local practice | 10 (35.7)  18 (64.3) | 6 (23.1)  20 (76.9) | 0.38 |
| Associated Immunosuppression regimen (%)   - MPA - everolimus - azathioprine - steroids | 15 (53.6)  7 (25.0)  1 (3.6)  24 (85.7) | 16 (61.5)  6 (23.1)  1 (3.8)  23 (88.5) | 0.59  >0.99  >0.99  >0.99 |
| Tacrolimus monotherapy (%)  Tacrolimus bitherapy with:   - steroids - MPA - everolimus   Tacrolimus tritherapy with:   - MPA and steroids - everolimus and steroids - azathioprine and steroids | 1 (3.6)  7 (25.0)  4 (14.3)  1 (3.6)  2 (7.1)  20 (71.4)  14 (50.0)  5 (17.9)  1 (3.6) | 0  6 (23.1)  3 (11.5)  1 (3.8)  2 (7.7)  20 (76.9)  14 (53.8)  5 (19.2)  1 (3.8) | >0.99  >0.99  >0.99  >0.99  >0.99  0.76  0.79  >0.99  >0.99 |
| Serum creatinine (µmol/L), median (IQR_25-75_)   - one year before conversion - at conversion - one year after conversion | 129 (101; 158)  139 (99; 150)  140; 109; 170) | 149 (99; 156)  132 (109; 151)  125 (99; 150) | 0.82  0.80  0.25 |
